# RETRACTION: TRIM29 as a Novel Biomarker in Pancreatic Adenocarcinoma

**DOI:** 10.1155/dim/9763697

**Published:** 2025-11-05

**Authors:** Disease Markers

RETRACTION: H. Sun, X. Dai, and B. Han, “TRIM29 as a Novel Biomarker in Pancreatic Adenocarcinoma,” *Disease Markers* 2014, no. 1 (2014), https://doi.org/10.1155/2014/317817.

The above article, published online on 22 April 2014 in Wiley Online Library (wileyonlinelibrary.com), has been retracted by John Wiley & Sons Ltd.

The retraction has been agreed following an investigation of the concerns raised by *Hoya camphorifolia* on PubPeer [1], which identified an instance of figure duplication.

Specifically, the microscopy images of SW1990 and BxPC3 cells presented in Figures 4a,b respectively, share unexpected similarities Figures 4A,C in [2], which denote HCT116 cells.

Further investigation revealed the following additional concerns:

- Figure 4a, panel ‘SW1990 - siTRIM29', shares unexpected similarities with Figure 4a, panel ‘BxPC3 - siTRIM29';
- Figure 4a, panel ‘BxPC3 - siTRIM29', shares unexpected similarities with Figure 4b, panel ‘BxPC3 - si-Scramble';
- Microscopy images reporting BxPC3 cells in Figures 4a,b share unexpected similarities with Figure 4c in [3], which denotes PANC-1 cells;
- Figure 4a, panel ‘BxPC3 - si-Scramble' shares unexpected similarities with Figure 3c, panel ‘SaOS2-NC – Invasion' in [4].

As a result of the investigation, the data and conclusions of this article are considered unreliable.

The journal was unable to reach the authors for response regarding these concerns.
